# *Hibiscus sabdariffa* Meal Improves Iron Status of Childbearing Age Women and Prevents Stunting in Their Toddlers in Northern Ghana

**DOI:** 10.3390/nu11010198

**Published:** 2019-01-19

**Authors:** Clement Kubreziga Kubuga, Hyokyoung Grace Hong, Won O. Song

**Affiliations:** 1 Nutritional Sciences Department, University for Development Studies, Tamale, Ghana; kubuga@yahoo.com; 2 Department of Statistics and Probability, Michigan State University, EL, MI 48824, USA; hhong@stt.msu.edu; 3 Food Science and Human Nutrition Department, Michigan State University, EL, MI 48824, USA

**Keywords:** iron deficiency, iron status, anemia, *Hibiscus sabdariffa*, reproductive age women, toddlers, Ghana

## Abstract

Globally, iron deficiency (ID) is the most common form of nutritional deficiency, particularly in young children and childbearing age women. ID can lead to stunting and impaired cognitive development in children, as well as adverse maternal health and birth outcomes. In this study, the efficacy of an alternative food-to-food fortification utilizing indigenous iron-rich food sources was investigated in a quasi-experimental study. Childbearing age women (15–49 years, intervention-Kassena Nankana West district: *n* = 60; control-Builsa North district: *n* = 60) and their toddlers (6–24 months) consumed *Hibiscus sabdariffa* leaf meals (HSM, 1.71 mg Fe/100 g meal) three times a week for 12 weeks during the dry/lean season in Northern Ghana. We found that feeding the HSM (1.9 kg/day) improved iron status of women of childbearing age with time (*p* = 0.011), and protected stunting among toddlers during the dry/lean season (*p* = 0.024), which is the period with the worst food and nutrition insecurity. Compared with the control group, the number of stunted toddlers declined in the intervention group.

## 1. Introduction

Worldwide, iron deficiency (ID) is the most common form of nutritional deficiency in children and childbearing age women [1,2,3,4]. In Ghana, iron deficiency and anemia are major problems [5,6,7,8,9,10], especially among women of reproductive age and children. A recent study indicated that 13.7% and 21.5% of women and children are, respectively, iron deficient [11]. Within Ghana, the three northern regions (“Northern belt”) had higher prevalence of anemia (women: 27.6%, children: 53.2%), iron deficiency (women: 21.5%, children: 39.6%), and iron deficiency anemia (IDA, women: 15.4%, children: 29.0%) compared to those in the southern regions for anemia (women: 23.3%, children: 32.3%), ID (women: 13.6%, children: 12.6%), and IDA (women: 9.2% children: 5.2%) [11]. The same study also noted that prevalence of stunting was highest in the Northern belt (25–30%). 

Consequences of IDA include poor maternal health, poor fetal growth [12,13], and adverse birth outcomes, such as growth retardation, stunting, and impaired cognitive development [14]. Globally, several approaches were implemented to counteract iron and other trace mineral deficiencies [15]. The measures included malaria control, fortification of food, supplementation of the minerals, and dietary diversity approaches. Developing countries with high prevalence of malaria present challenges associated with the fortification of food and supplementation of elemental iron, such as access by rural households, cost, and sustainability of the products [15]. To overcome the complications of malaria (such as parasitemia) and inflammation in improving and assessing iron status, World Health Organization (WHO) recommends the use of serum concentration of soluble transferrin receptor (sTfR) in monitoring iron status in developing countries. Compared to serum ferritin, sTfR is a sensitive biomarker for iron status and is less affected by infection and inflammation [16].

In Ghana, numerous costly supplementation and food fortification programs were adopted to counteract iron and other micronutrients deficiencies [17,18,19]. Those programs include iron and folic acid supplementation for pregnant women, national vitamin A supplementation for children under five years and pregnant and lactating women, refined oils and wheat flour fortification, and universal salt iodization policy [11,17,18,19]. However, to the best of our knowledge, efficacies of these individual programs implemented at the national level have not yet been monitored or reported in the midst of the high prevalence of anemia.

In developing countries like Africa, ID often accompanies nutritional deficiencies of many other micronutrients and proteins due to monotonous and plant-based staple food intake with limited nutrient-rich animal sourced of foods. Sustainable, food-based, practical, and cost-effective, long-term, alternative solutions to prevent ID at the population level in Africa [7,10] are urgently needed to be explored. Little data on food composition and iron content of indigenous foods exists in sub-Saharan African countries, including Ghana. The aim of this study was to investigate the efficacy of native *Hibiscus sabdariffa* leaf meal (HSM) in improving iron status of reproductive age women and their toddlers, as well as stunting in the toddlers through a community-based feeding intervention during dry/lean season. 

## 2. Materials and Methods 

### 2.1. Study Design

This community-based 12-week feeding trial (clinical trials.gov ID: NCT03754998) was planned in a quasi-experimental design with the primary focus of assessing and improving iron status of dyads, determined by hemoglobin and sTfR. Baseline measurements (T1) were obtained prior to the intervention. Follow-up measurements (T2 and T3) were obtained six weeks and 12 weeks after T1, respectively. At the time of planning the current community intervention study (2015/2016), the PIs did not see the study fit under the classical definition of clinical trials that include any novel products (drugs, foods, instruments), but the definition has expanded since.

**Figure 1:**
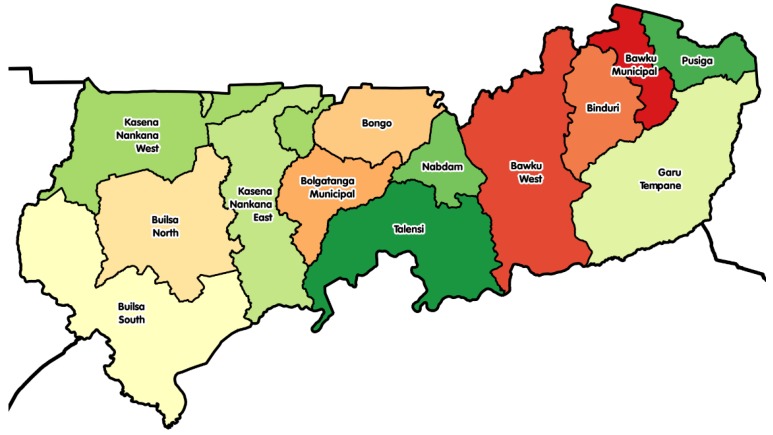
A map of the Upper East region showing its districts.

**Study site:** This study was carried out in two districts in Upper East Region in Ghana (Figure 1): Kassena Nankana West (KNWD) and Builsa North (BND) districts. The capitals of these districts are about 25 km away from each other, are linked with a single paved road via the Kassena Nankana Municipality, and about 42 km away from the regional capital Bolgatanga. The study area has a typical savannah woodland vegetation characterized by short, scattered, drought-resistant trees and grass. The study area has two main seasons: Dry season (October–April/May), characterized by high temperatures, and rainy season (May/June–September). People in this area are mostly subsistence farmers. The study area is malaria endemic with peaks at the end of the rainy season when 45% of households are food insecure [20]. These districts were among the top five food insecure districts in the region. The selected sites for this study were two communities (Sakaa and Chania) in KNWD and three communities (Chuchuliga-yipaala, Azoayeri, and Awulansa) in BND. The study sites were selected based on the inclusion criteria of having a functional borehole (water source) throughout the dry season, existing women groups, and access to Community-Based Health Planning and Services (CHPS) compounds, sizeable number of mother and young children (6–23 months) dyads for good sampling frames, and community health nurses who were willing to work with researchers between May and August 2016. Researchers’ previous experience with those communities facilitated the community entry process. 

### 2.2. Study Subjects and Selection

The study subjects, women 15–49 years and their children 6–23 months, were recruited in May 2016. The dyads were drawn from selected districts using community-based birth registers at CHPS compounds kept by community health nurses stationed in these communities or by community health volunteers. The health volunteers are community members. Announcements through the community chiefs/leaders were then made in the respective communities for all women with children under five years of age to meet at their respective community health centers. The research team briefed the women and the community leaders on the study. Names that were shortlisted from the birth registers were read out. All dyads including those who were not in the register, but obtained from the health volunteers who knew almost everybody in their catchment areas, were contacted and checked on their willingness to participate in the study through the community health volunteers and community health nurses. Finally, verbal consent was sought from spouses of the women who were willing to be part of the study. 

A total of 120 dyads (60 mother/child dyads each for intervention and control group) were drawn from the two districts and agreed to participate in the study. Optimal design software (version 3.01) was used to calculate the minimal sample size of 100 dyads (50 dyads each for intervention and control groups) with a power of 80%, significance level of 5%, coefficient of determination of 65%, minimum detectable effect of 0.33 of diet on hemoglobin change, and 20% attrition rate. 

We had three sets of twins, one in intervention group and two in the control group in the study. We had a 7% attrition rate in intervention group. Two dyads relocated before baseline data collection and two relocated in the sixth week. All relocations were to either join a spouse or the entire nuclear family was migrating for farming purposes.

### 2.3. Intervention-HSM

Feeding trial: The participating dyads in intervention communities were invited to consume veo soup/meal (HSM) three times a week and were provided a weekly supply of iodized salt (450 g) for the household usage.

Veo soup modified: The veo soup/meal is a local Ghanaian soup/meal mainly made of *Hibiscus Sabdarifa* leaves. It is a soup when it is prepared a bit watery and consumed with ‘tou zaafi’ (millet- or corn-based cooked paste). It is a meal when prepared thick and eaten by itself. The *Hibiscus sabdariffa* leaf meal (HSM) used in the present study was made of 18 kg *Hibiscus sabdariffa* leaves, 8 kg groundnut, 1.1 kg *dawadawa* (fermented African locust beans), 3 kg dried fish, plus 0.045 kg iodized salt, cooked with about 23 L (23 kg) water to yield 52.5 kg HSM. Dried fish or meat is a commonly added ingredient of HSM for people of high socioeconomic status, especially in the cities. The diet is named as HSM, as diets are usually labelled per their major ingredients.

The ingredients of the HSM were provided to the women, who prepared the meals themselves. This helped in building trust and avoiding any forms of suspicion during supervision of all cooking and feeding activities. In each community, groups of ten women took turns to share the cooking activities, washing of bowls, and making water available for cooking. Three women in each community were trained on food weighing/measurement to conform to our standardized recipe. It took 45–60 min to cook a batch of the meal, which was then served (1.5 kg/woman) to all women and their children (0.5 kg/child) separately. The women and toddlers were given two separate bowls, so that the researcher could monitor each person’s consumption. All dyads were encouraged to consume to their satisfaction by requesting additional servings. The meal intake was measured by the researcher (CK) and trained women by the differences between the quantities served minus leftovers. The women and toddlers consumed, on average, 1.9 kg and 0.4 kg, respectively.

The food composition analysis of the final HSM was carried out by Great Lakes Scientific, Inc. (Stevensville, MI, USA). Our standardized HSM (per 100 g) contained 1.7 mg iron, 6.6 mg protein, 4.6 mg fat, 82.6% water, and 2.7% ash. 

### 2.4. Measurements and Data Collection

Questionnaire/interview: A questionnaire on food intake frequency and 24 h food intake recall was administered at baseline and at the end of the study. At baseline, information on sociodemographic characteristics was also taken. 

Malaria status was screened by the researcher at baseline, midpoint, and at the endpoint for all research participants using Rapid Malaria Diagnostic cassettes (Lot: 05CDB050DA. Standard Diagnostic, Inc. Republic of Korea.). Malaria screening is based on the presence of Histidine-Rich Protein-2 (HRP-II) in whole blood. HRP-II is known to be specific to *Plasmodium falciparum*, which causes more than 90% of malaria cases in Ghana [21]. The sensitivity and specificity of cassettes were 95% and 99.5%, respectively. All participants who were screened positive for malaria were referred to their respective CHPS compounds for treatment. In most instances, the community health nurses, who were in charge of compounds and also part of our research team, provided medicine to toddlers on site and women upon presenting their health insurance identification. Anemic individuals were also referred for treatment as ethically required, and potential interference or complication in intervention outcomes were handled during data analysis. Referred individuals were part of the study to the end, thus included in the data analyses. We modeled the Difference-in-Differences with continuous outcome measures (Hb and sTfR) and repeated measures ANOVA using the GLM procedure. This was done for ID and iron sufficient (IS) individuals at baseline and subsequently combined IS and ID. These analyses indicated non-significance. Interestingly, reporting rates of adequate and inadequate iron status is integral to our study analysis, so estimates of absolute risk differences are desirable. We used PROC GENMOD, a marginal population-level model (General Estimating Equations, or GEE) to generate these estimates in a binomial model using the identity link [22,23].

Anthropometry: Participants’ weights and heights were measured at baseline, midpoint, and at the endpoint, according to standard procedures [24]. Electronic scale (Serial number 5874030154862, Model 874 1321009. Seca gmbh & co kg, 22089 Hamburg, Germany.) was used to measure weights to the nearest 0.1 kg and height measured by Seca 217 (Seca gmbh & co kg, 22089 Hamburg, Germany.).

Biochemical measurements: At baseline and endpoint, 5 mL whole blood was withdrawn into silica-coated serum separator vacutainers (Lot: 20140618, Anhui Kangning Industrial (Group) Co. Ltd, Tianchang City, China) and held at ambient temperature before and during transportation. Serum was separated using a centrifuge (Hettich) at 500× *g* for 5 min at room temperature. Separated serum was aliquoted, kept frozen at −18 °C (Thermo Fisher Scientific) at NHRC, then transported on dry ice to MDS Lancet laboratories in South Africa for serum transferrin receptor analysis. Analysis was done using Tina-quant Soluble Transferrin Receptor 80 tests, Roche/Hitachi cobas c 311, cobas c 501/502. Hemoglobin levels were measured in the field using HB 201 analyzer according to prescribed procedures. At midpoint, only hemoglobin levels were measured. 

Nutrient measurement: Iron concentrations of HSM*, Hibiscus leaves, dawadawa, Amani,* and *groundnut* were obtained by removing the organic content of samples in a high temperature muffle furnace. The resulting ashes were diluted in acid and absorbance was read by Atomic Absorption Spectrometry (3110 Perkin Elmer Atomic Absorption Spectrometer with a Hollow Cathode Calcium/Magnesium Lamp at 285.5 nm). Analyses were carried out by standard procedures for iron (AOAC 985.35), protein (AOAC 928.08II), fat (AOAC 925.12), moisture (AOAC 950.46A), and ash (AOAC 923.03). Analytical work was carried out by Great Lakes Scientific Inc. Stevensville, MI, USA. Proportion of iron from the various ingredients of HSM can be found in Table S1.

### 2.5. Statistical Analysis

Data analyses were carried out by SAS 9.4 (SAS institute, Cary, NC, USA). Characteristics of participants were described using frequency distributions. Comparison of participants’ characteristics between intervention and control groups was done using Chi-square statistics and Student t-test for categorical and continuous variables, respectively. 

Dependent variables of interest were linear growth (Stunting: HAZ < −0.2) of toddlers, and iron status (ID: sTfR > 4.40 and > 2.85 ug/L in women and toddlers Lab reference; anemia: Hb < 12 g/dL; IDA: concurrent presence of anemia and ID). Linear growth and iron status (measured by sTfR) were measured only at T1 and T3. T1, T2, and T3 are referred to herein as the time variable. Multiple logistic regression was fitted to identify the risk factors of iron status (ID), which is a binary variable. Since the response is discrete and correlated within the same subject, to account for dependencies, we modeled the relationship between the binary outcome and predictors using generalized estimating equations (GEEs) in PROC GENMOD. Control variables are household wealth index, marital status, sex of household head, mother's age, child's age, malaria status, participation in household decision-making, and the number of children and adults in household, which are measured at baseline study. Chi-square statistics was used to compare linear growth, IDA, and anemia status measured by hemoglobin levels in control and treatment groups.

We compared the intra group hemoglobin levels using PROC MIXED, as we were interested in comparing or modeling means of hemoglobin levels. Statistical analysis for hemoglobin was done in twofold: 1) Analysis using nonanemic (Hb ≥ 12 g/dL) participants at baseline, and 2) analysis with anemic (Hb < 12 g/dL) plus nonanemic (Hb ≥ 12 g/dL) participants combined. It is worth noting that for iron status using sTfR, we had 58 samples at baseline in intervention, but one subject was excluded in our analysis due to inadequate sample volume for such analysis. At the endpoint, we had 53 results, which included endpoint results for the one missing at baseline, explaining the discrepancies in Table 1 and Table 2. The combined number of drop outs and missing data (12%) is less than the anticipated attrition rate of 20%. Based on the attrition rate of 12%, the final data analysis is not expected to be affected. Additionally, participants with missing variables were not significantly different from the other participants.

Ethics*:* Research procedures were in accordance with the Michigan State University’s Institutional Review Board and Navrongo Health Research Centre (NHRC) Institutional Review Board (IRB) in Ghana

## 3. Results

Sociodemographic characteristics of childbearing age women and their toddlers in both districts is summarized in Table 3. For several variables, no significant differences between the two districts found (participation in household decision-making, wealth index, household head’s sex, living with animals in same compound, number of adults and children under five years in household, number of child’s siblings, ages of woman, child, and household head), except for women’s occupation, religion, ethnicity, and iodine content in household salt. All the women who reported other forms of occupations were also engaged in farming in the wet season. 

Health indicators of mother and toddler dyads (Table 1: Weight, height, serum transferrin receptor level, hemoglobin level, malaria status, anemia and iron status) did not differ between the intervention vs. control groups, except toddlers’ anemia status and height. Of the toddlers’ nutritional indicators, *Z*-scores for HAZ, but not WHZ, differed between the two groups when examined as a continuous variable. However, the differences disappeared when proportion of stunted and non-stunted was compared between the groups. Anemia, ID, and IDA prevalence did not differ significantly between intervention and control groups across time except for anemia and IDA in toddlers at baseline. There was significant difference between groups for stunting at endpoint (*p* = 0.024).

The difference-in-difference of sTfR and hemoglobin levels at endpoint among mothers who were ID and IS at baseline did not differ between the groups, as summarized in Table 2. When ID and IS individuals were combined, there was still no significant difference between the groups. Iron status (ID and IS), however, indicate a significant lower change of 0.3% in iron deficiency prevalence in intervention group than in the control group, hinting that the intervention benefit may have helped improve iron status. Similar analysis is done in Table 4.

Table 4 indicates endpoint comparison of iron status (combined individuals who were IS (≤4.40 μg/L) and ID (>4.40 μg/L) at baseline) for women. Intervention group appears to be protective of iron deficiency with time (*β* = −0.32, *p* = 0.011) after adjusting for household wealth index, marital status, sex of household head, mother's age, child's age, malaria status, participation in household decision-making, and the number of children and adults in household. This could suggest that we needed a longer duration to enable us establish differences between groups. Households with male heads are at a higher risk of iron deficiency (coefficient = 1.66, *p*-value = 0.045) as compared to households with female heads.

On hemoglobin (Table 5) for women, there were no significant differences between group means. However, changes within groups were observed. Intervention group had some significant variations (T3–T1: Mean ∆ = 0.24 g/dL, *p*-value = 0.0987; T3–T2: Mean ∆ = −0.06 g/dL, *p*-value = 0.0442; and T2–T1: Mean ∆ = 0.06 g/dL, *p*-value = 0.6712), but not in the control group (T3–T1: Mean ∆ = 0.05 g/dL, *p*-value = 0.7065; T3–T2: Mean ∆ = 0.11 g/dL, *p*-value = 0.6479; and T2–T1: Mean ∆ = −0.06 g/dL, *p*-value = 0.0987). After adjusting for covariates (household wealth index, marital status, sex of household head, mother's age, child's age, malaria status, time*group interaction, empowerment, number of children and adults in household), T3–T1 (*p* = 0.079) and T3–T2 (0.0824) became marginally significant in intervention group. Changes within control group remain insignificant after adjustment. Women and toddlers in the intervention consumed, on average, 1.9 kg and 0.4 kg, respectively, of HSM per day.

## 4. Discussion

Iron fortification of foods has long been implemented worldwide in an effort to control ID and IDA prevalence. The approach has been said to be sustainable, practical, and cost-effective in the long term at the population level. However, iron fortification of foods showed limited success in Africa [7,10], as fortification may not reach people at greatest risk for nutritional ID since they depend on subsistence farming with little access to processed food items [25]. Global approaches to counteract iron deficiencies have to be diverse and robust to address specific needs of regions, populations, and cultural settings. Dietary approaches, such as food-to-food fortification, can be a promising approach to improve trace mineral intake, especially in situations of low dietary trace mineral intake and bioavailability from monotonous diets based on a small number of staple plant foods [26], as is the case in most developing countries. The fundamental challenge is often the unknown efficacy of these food-to-food fortifications to improve iron status. Our study sought to test the potential of indigenous *Hibiscus sabdariffa* meal to improve iron status in mother-child dyad and linear growth in toddlers.

The results of our study suggest that indigenous *Hibiscus sabdariffa meal* improves iron status of women of child-bearing age with time and could be protective of stunting among toddlers during the dry/lean season. Lean season in developing countries is widely known to affect food and nutrition security [27], mitigating the effect of nutrient deficiencies in this period is thus very timely and crucial. Our findings could be one of the needed solutions to the high prevalence of iron deficiency and anemia prevalence, especially in the dry season in Northern Ghana. 

This study is among the very few studies in Ghana showing the potential of a food-based approach to improve iron status without using biosynthetically incorporated elemental iron. A modest increase in iron status, as indicated by sTfR, was observed in a food study trial using animal-source food in rural Vietnam [28]. In another fold, cowpea-based food containing fish meal and served with a vitamin C–rich drink improved hemoglobin concentration, but not ferritin levels [29]. Consumption of heme iron-rich foods (goat meat and liver) and enhancers (orange juice and other fruit juices) of non-heme absorption decreased anemia and iron deficiency anemia, but not ferritin levels, in Burkinabe preschoolers [30]. These findings point to the difficulty involved in testing the efficacy of food-to-food fortification. Interestingly, our study indicates significant improvement with time, suggesting that a longer duration is required to reveal clear cut differences between groups. Time and intervention group interaction effect shows statistically significant (*β* = −32, *p* = 0.011) differences between groups. This further points to the role of time and the need for a longer duration for the study. Similarly, a significant lower change of 0.3% in iron deficiency prevalence in intervention group was observed, hinting that the intervention benefit may have helped improve iron status at the study endpoint.

We had similar observations in hemoglobin levels. The velocity of change was only prominent in the intervention group, with the key change between the midpoint to the endpoint (*p* = 0.044, Adjusted *p* = 0.082). This suggests a need for a longer duration for the trial to clearly elucidate the impact of the diet (*Hibiscus sabdariffa* meal). 

At baseline, nearly all the toddlers were anemic and ID and were referred for treatment. Analysis among IS toddlers was not feasible due to the small sample size. Similar comparison, as in the case of the women, could not be done, as such the effect of the meal could not be established among the toddlers. This is a very important flaw of the study design, but important to follow the ethical requirement and conflict. ideally, the toddlers should have been treated and rolled on to the program, but constraints beyond our control did not support this approach.

On the other hand, stunting levels were not significantly different between groups at baseline but increased by 5.1% and decreased by 1.4% in control and intervention groups, respectively. The increase may be common, as the dry/lean season advances to all children, but the intervention protected the adverse effect of dry/lean on children’s stunting. Growth in this case may not be linked to the iron content but the additional meals provided by feeding interventions. It is known that supplementation of such single nutrients as protein, zinc, iron, copper, iodine, and vitamin A can benefit linear growth in these chronically malnourished children. Our findings suggest that growth is limited by multiple and simultaneous deficiencies in many nutrition-insecure populations [31].

The strengths of our study were the use of control and intervention groups to elucidate the effect of the intervention. The use of indigenous staple foods makes the findings relevant and practical to impacting nutritional status of vulnerable populations. Furthermore, to the best of our knowledge, our study is the first of its kind in the research settings to strictly use only indigenous meals without using elemental iron to improve iron and nutritional status. This study is not without limitations/challenges. We recognized that not measuring inflammatory markers could have influenced our results. To minimize the impact of infections and inflammation, we used transferrin receptor, as recommended by WHO. We suggest measurement of various markers of iron and inflammation be done in future studies. The timing for our study was short and might have impacted on our results, we suggest a 6—12 months study in future research works. Not conducting a bioavailability study on our diet is another limitation that needs to be addressed in subsequent studies. Complete treatment of all individuals with anemia especially in the case of the toddlers before rolling them into the program would give the full effects of the prescribed intervention.

## 5. Conclusions

We demonstrated the potential benefits of indigenous *Hibiscus sabdariffa* meal in improving iron status of women of childbearing age with time, and its protectiveness against stunting among toddlers during prolonged dry/lean season when prevalence of food and nutrition insecurity are highest. Our findings present one of the urgently needed solutions to tackling iron deficiency and stunting among rural populations by policymakers, health and nutrition educators, as well as all stakeholders in the health sector in Ghana. In tackling iron status among women and linear growth in resource poor settings, modifying indigenous diets with locally available plant- and animal-based ingredients could give the needed or desired outcomes. Though our findings look promising, we recognize the need for further research on the efficacy of the *Hibiscus sabdariffa* meal/soup to improve iron status and linear growth.

## Figures and Tables

**Table 1:** Health indicators of childbearing age women (15–49 years) and their toddlers (6–23 months) in intervention and control groups across time.

Variable	Intervention		Control		
Women					
Baseline (T1)	*N*	Mean ± SD	*N*	Mean ± SD	*p*-value
women height (cm)	58	160.3 ± 6.6	60	161.7 ± 5.6	0.217
women weight (kg)	58	57.1 ± 9.7	60	57.3 ± 7.6	0.888
women Hb (g/dL)	58	11.8 ± 1.3	60	11.8 ± 1.2	0.775
women sTfR (μg/L)	57	5.1 ± 3.1	60	4.7 ± 1.6	0.320
	Status	*N* (%)	*N* (%)	*X* ^2^	*p*-value
women ID (sTfR)	deficient	28 (49.1)	28 (46.7)	0.07	0.790
women IDA	anemic	12 (21.0)	19 (31.7)	1.69	0.194
women anemia (Hb)	Anemic	27(46.5)	35(58.3)	1.64	0.200
Women malaria	Positive	7(12.1)	5(8.3)	0.50	0.502
**Week 6 (T2)**					
women anemia (Hb)	Anemic	27(51.9)	26(44.8)	0.55	0.457
women malaria	Positive	20(39.2)	18(31.0)	0.80	0.371
**Week 12 (T3)**					
women ID (sTfR)	deficient	18(44.0)	20(35.7)	0.04	0.848
women IDA	anemic	11(20.2)	13(23.2)	0.10	0.757
women anemia (Hb)	anemic	24(44.4)	30(51.7)	0.59	0.441
women malaria	Positive	11(20.0)	13(22.4)	0.10	0.754
Toddlers					
Baseline (T1)	*N*	Mean ± SD	N	Mean ± SD	*p*-value
HAZ	59	0.3 ± 1.3	62	−0.3 ± 1.3	0.009
WHZ	59	−1.2 ± 1.3	62	−0.9 ± 1.3	0.294
toddlers Hb (g/dL)	59	9.4 ± 1.7	62	9.0 ± 1.3	0.171
Toddlers sTfR (μg/L)	58	9.1 ± 5.1	62	9.7 ± 5.3	0.528
	Status	*N* (%)	*N* (%)	*X* ^2^	*p*-value
toddlers ID (sTfR)	anemic	56(96.6)	61(98.4)	0.40	0.520
children IDA	anemic	47(81.0)	59(95.2)	5.80	0.016
toddlers anemia (Hb)	anemic	50(84.8)	60(96.8)	5.29	0.021
Children malaria	Positive	4(6.8)	6(9.8)	0.40	0.545
Stunting (HAZ)	Stunted	3(5.1)	7(11.3)	1.50	0.215
**Week 6 (T2)**					
toddlers anemia (Hb)	anemic	49(94.2)	58(96.7)	0.40	0.534
children malaria	Positive	20(38.5)	7(11.7)	10.93	0.001
		**Week 12 (T3)**			
toddlers ID (sTfR)	deficient	58(100)	56(96.6)	2.04	0.154
children IDA (sTfR)	anemic	42(72.4)	46(79.3)	0.75	0.386
toddlers Anemia (Hb)	anemic	43(78.2)	49(42.6)	0.20	0.641
children malaria	Positive	18(32.7)	11(18.3)	3.15	0.076
Stunting (HAZ)	stunted	2(3.64)	10(16.39)	5.08	0.024

Iron deficiency (ID), defined by sTfR > 4.40 μg/L and >2.85 μg/L in women and toddlers, respectively. Anemia, defined by Hb < 12 g/dL and Hb < 11 g/dL, in women and toddlers respectively. Iron deficiency anemia (IDA), defined by concurrent presence of anemia and ID. Positive malaria: Have malaria. HAZ: Height-for-age *z*-score. WHZ: Weight-for-age *z*-score.

**Table 2:** Endpoint comparison of iron status among childbearing age women (15–49 years) in intervention and control groups.

Variable	*F*-value	*p*-value
D-I-D		
sTfR ≤ 4.40	0.00	0.996
sTfR > 4.40	0.17	0.684
sTfR-combined	0.16	0.693
Hb ≥ 12.0 g/dL	0.03	0.859
Hb < 12.0 g/dL	2.49	0.120
Hb-Combined	0.70	0.406
D-I-D (ID status)	estimate	*p*-value
Intervention	−0.003	0.032
Control	Reference	

ID: sTfR > 4.4 μg/L. D-I-D: Difference in difference. sTfR-combined: Individuals with Hb ≥ 12.0 g/dL plus Hb < 12.0 g/dL. sTfR-combined: Individuals with sTfR ≤ 4.0 μg/L plus sTfR > 4.0 μg/L.

**Table 3:** Demographic characteristics of childbearing age women (15–49 years) and their toddlers (6–23 months) in intervention and control groups at baseline.

Variable		Intervention		Control		*p*-value
		*N*	Mean ± SD	N	Mean ± SD	
Women age (years)		58	26.7 ± 6.7	60	26.1 ± 5.4	0.598
Toddlers age (mo)		59	14.1 ± 5.1	62	12.6 ± 5.2	0.119
Household head’s age		58	47.0 ± 16.9	60	42.7 ± 15.1	0.149
Number of household adults		58	4.6 ± 3.3	60	3.7 ± 1.4	0.051
# Siblings in household		59	1.4 ± 1.6	62	1.9 ± 1.5	0.092
# children < 5 years		58	1.0 ± 1.2	60	1.1 ± 1.2	0.708
Variable		*N*	%	N	%	X^2^ (*p*-value)
Occupation of women	Farmer	31	53.5	50	83.3	15.3 (0.002)
	Handy works	5	8.6	5	8.3	
	Housewife	6	10.3	2	3.3	
	Trader	16	27.6	3	5.0	
Household head sex	Male	54	93.1	56	93.3	0.00 (0.960)
	Female	4	6.9	4	3.4	
Wealth index	Lower	19	32.8	20	33.3	0.02 (0.991)
	Middle	20	34.5	20	33.3	
	Upper	19	32.7	20	33.3	
Decision-making	Low	11	20.0	9	15.0	1.2 (0.545)
	Average	27	46.6	34	56.7	
	Above	20	34.5	17	28.3	
Household iodize salt	Non-users	46	80.7	59	98.3	9.8 (0.002)
	Users	11	19.3	1	1.7	
Iodine level in household salt	<15 ppm	52	91.2	60	100.0	5.5 (0.019)
	≥15 ppm	5	8.8	0	0.0	
Ethnicity of women	Builsa	2	3.4	56	90.3	92.3 (< 0.000)
	Kassena	55	93.2	5	8.1	
	Nakani/Fulani	2	3.4	1	1.6	
Religion	Christian	34	57.6	60	96.8	27.0 (< 0.000)
	Muslim	9	15.3	0	0.0	
	Traditionalist	16	27.1	2	3.2	
Animals live in household	Yes	0 51	87.9	58	96.7	3.2 (0.074)
	No	7	12.1	2	3.3	

Traditionalist: Practitioners of African traditional religion; Decision-making: The degree in which women participate in household decision-making; Wealth index: Household cumulative living standard measured using household assets. #: Number.

**Table 4:** Comparison of iron deficiency among childbearing age women (15–49 years.) in intervention and control groups across time.

Parameter	Estimate	SE	95% CI		*p*-value
Intervention Group	0.17	0.52	−0.85	1.19	0.749
Control group	Ref				
Male household head	1.66	0.82	0.04	3.27	0.045
Female household head	Ref				
Malaria absent	0.15	0.39	−0.61	0.90	0.701
Malaria present	Ref				
Lower wealth index	0.52	0.47	−0.40	1.44	0.269
Middle wealth index	0.45	0.41	−0.34	1.25	0.265
Upper wealth index	Ref				
Married	−0.23	0.93	−2.04	1.59	0.806
Single	−1.22	1.13	−3.43	1.00	0.282
Divorce/separated	Ref				
Low decision making	−0.66	0.54	−1.73	0.41	0.227
Average decision making	−0.79	0.46	−1.69	0.10	0.081
Above average decision making	Ref				
Time and intervention group interaction effect	−0.32	0.13	−0.57	−0.07	0.011
Time and control group interaction effect	−0.25	0.15	−0.56	0.05	0.097
BMI	0.37	0.33	−0.27	1.01	0.256
Women age (years)	−0.02	0.03	−0.09	0.04	0.448
Toddlers age (months)	−0.05	0.04	−0.12	0.01	0.121
Number of household Adults	−0.09	0.11	−0.29	0.12	0.426
# toddlers < 5 years in household	−0.04	0.18	−0.38	0.30	0.819

Adjusted: Household wealth index, marital status, sex of household head, mother's age, child's age, malaria status, #: number of, participation of women in household decision-making, number of children and adults in household.

**Table 5:** Hemoglobin levels of child-bearing age women and the effects of *Hibiscus sabdariffa* meal consumption on indicators across study period.

	**Nonanemic and anemic-referred mothers**					
Variable/Time	Intervention			Control		
	Mean	*p*-trend	Adj *p*-value	Mean	*p*-trend	Adj *p*-value
Hb T1	11.84	0.103		11.77	0.704	
T2	11.78			11.87		
T3	12.08			11.82		
Group Hb	11.96			12.05		0.690
	Mean ± SE	*p*-value	Adj *p*-value	Mean ± SE	*p*-value	Adj *p*-value
T1–T3 Hb	−0.24 ± 0.15	0.099	0.079	−0.05 ± 0.13	0.707	0.567
T1–T2 Hb	0.06 ± 0.15	0.671	0.954	−0.11 ± 0.13	0.403	0.347
T2–T3 Hb	−0.30 ± 0.12	0.044	0.082	0.06 ± 0.13	0.648	0.692
**Nonanemic mothers at baseline**						
T1–T3 Hb	−0.01 ± 0.21	0.789	0.966	0.12 ± 0.20	0.634	0.550
T1–T2 Hb	0.22 ± 0.23	0.148	0.342	0.13 ± 0.21	0.690	0.540
T2–T3 Hb	−0.23 ± 0.22	0.234	0.292	−0.01 ± 0.20	0.933	0.971
	Mean	*p*-trend		Mean	*p*-trend	
T1 Hb	12.44	0.515		12.68	0.785	
T2 Hb	12.22			12.55		
T3 Hb	12.45			12.56		
Group Hb	12.37			12.60		0.983

T1: Baseline, T2: Midpoint, T3: Endpoint. Adjusted: Household wealth index, marital status, sex of household head, mother's age, child’s age, malaria status, participation of women in household decision-making, number of children and adults in household. Note: The women and toddlers in intervention consumed, on average ,1.9 kg and 0.4 kg, respectively of hibiscus meal per day.

## Supplementary Materials

The following are available online at http://www.mdpi.com/2072-6643/11/1/198/s1, Table S1: Nutrient composition of HSM and its ingredients.
