# Supplementary material for: Hibiscus sabdariffa Meal Improves Iron Status of Childbearing Age Women and Prevents Stunting in Their Toddlers in Northern Ghana
Source: Nutrients. 2019 Jan 19;11(1):198. doi: 10.3390/nu11010198 (PMC6357016; doi:10.3390/nu11010198)
Supplement: Supplementary file 1 [file nutrients-11-00198-s001.pdf]

Table S1: Nutrient composition of HSM and its ingredients

Item	Iron (mg/100mg)	Protein (mg/100mg)	Moisture (%)	Fat (mg/100gm)	Ash (%)
HSM (meal)	1.71	6.58	82.6	4.6	2.7
Hibiscus Leaves	19.3	-	-	-	-
Dawadawa	8.43	-	-	-	-
Amani (dried fish)	22.91	-	-	-	-
Groundnut (pea nut)	2.09	-	-	-	-

HSM: Hibiscus Sabdariffa Leaves Meal. Dawadawa – Fermented African locust beans. Analysis done by Great Lakes Scientific, Inc .(www.glslab.com). Meal iron source/100mg: Hibiscus leaves: 0.63mg, Dawadawa: 0.27mg, Amani: 0.74mg, Groundnut: 0.07mg
